# Genome wide association study on feed conversion ratio using imputed sequence data in chickens

**DOI:** 10.5713/ajas.18.0319

**Published:** 2018-10-26

**Authors:** Jiaying Wang, Xiaolong Yuan, Shaopan Ye, Shuwen Huang, Yingting He, Hao Zhang, Jiaqi Li, Xiquan Zhang, Zhe Zhang

**Affiliations:** 1 Guangdong Provincial Key Lab of Agro-Animal Genomics and Molecular Breeding, College of Animal Science, South China Agricultural University, Guangzhou, 510642, China

**Keywords:** Genome-wide Association Study (GWAS), Imputed Whole Sequence Data, Feed Conversion Ratio, Chicken

## Abstract

**Objective:**

Feed consumption contributes a large percentage for total production costs in the poultry industry. Detecting genes associated with feeding traits will be of benefit to improve our understanding of the molecular determinants for feed efficiency. The objective of this study was to identify candidate genes associated with feed conversion ratio (FCR) via genome-wide association study (GWAS) using sequence data imputed from single nucleotide polymorphism (SNP) panel in a Chinese indigenous chicken population.

**Methods:**

A total of 435 Chinese indigenous chickens were phenotyped for FCR and were genotyped using a 600K SNP genotyping array. Twenty-four birds were selected for sequencing, and the 600K SNP panel data were imputed to whole sequence data with the 24 birds as the reference. The GWAS were performed with GEMMA software.

**Results:**

After quality control, 8,626,020 SNPs were used for sequence based GWAS, in which ten significant genomic regions were detected to be associated with FCR. Ten candidate genes, ubiquitin specific peptidase 44, leukotriene A4 hydrolase, ETS transcription factor, R-spondin 2, inhibitor of apoptosis protein 3, sosondowah ankyrin repeat domain family member D, calmodulin regulated spectrin associated protein family member 2, zinc finger and BTB domain containing 41, potassium sodium-activated channel subfamily T member 2, and member of RAS oncogene family were annotated. Several of them were within or near the reported FCR quantitative trait loci, and others were newly reported.

**Conclusion:**

Results from this study provide valuable prior information on chicken genomic breeding programs, and potentially improve our understanding of the molecular mechanism for feeding traits.

## INTRODUCTION

Increasing the productivity of livestock species and minimizing their environmental impact are the major goals in the livestock husbandry [1]. Improving feed efficiency would assist in meeting these challenges because feed consumption is about 70% of the total costs in livestock production [2]. Previous studies demonstrated that genetic selection could account for 85% to 90% of phenotypic improvement and plays a predominant role in underlying genetic architecture of increasing feed efficiency, while nutrition and management only explained 10% to 15% of phenotypic improvement [3]. Feed conversion ratio (FCR) is the most widely used measurement of feed efficiency by indicating how much feed mass livestock converts into the desired output. Up to now, a total of 32 quantitative trait loci (QTLs) associated with FCR have been reported. However, most QTLs are mapped with microsatellites markers by using linkage analysis [4] and genome scans [5] resulting in low resolution.

Compared with previous studies, genome-wide association studies (GWAS) can accurately identify genes involved in economically important traits of cattle [6], pigs [7], and chickens [8]. With the reduction of sequencing costs and rapid development of imputation technology, GWAS is the preferred option for such analyses. Using imputed whole genome sequence data, GWAS could take full advantage of all markers and detect variants associated with interesting traits, without being affected or influenced by the linkage disequilibrium between single nucleotide polymorphisms (SNPs) and the underlying genes [9]. These observations suggest that whole genome sequence data is an effective pipeline to enhance the power of GWAS.

The objective of this study was to identify candidate genes associated with FCR via GWAS in a Chinese indigenous chicken population using sequence data imputed from a SNP panel.

## MATERIALS AND METHODS

### Animals

The study population was obtained from a Chinese indigenous chicken breed that has been maintained for 25 generations by the Wens Nanfang Poultry Breeding Co. Ltd (Yun Fu, Guangdong, China). We obtained 435 male birds from the 25th generation, which were generated by 30 males and 360 females from the 24th generation and were reared with the same recommended nutritional and environmental conditions. The FCR was calculated according to recorded data during the feeding trial. For more details about this population, please refer to Zhang et al [10].

### Chip data and imputed sequences data

All of 435 male chickens from the 25th generation and 15 sires of the 24th generation were selected for genotyping. The genomic DNA from the 450 birds was extracted from blood samples using the NRBC Blood DNA Kit (Omega Bio-Tek, Norcross, GA, USA) according to the manufacturer’s instructions, and DNA samples were analyzed for genome DNA concentration and integrity. DNA samples of the appropriate quality were genotyped using 600K Affymetrix Axiom HD chicken genotyping array, which contains 580,961 SNPs distributed on 28 autosomes, two linkage groups (LGE64 and LGE22C19W28_E50C23), and two sex chromosomes. Then, twenty-four key individuals were selected for sequencing from the 435 birds and their 15 sires based on a strategy of maximizing the expected genetic relationship, while maximizing the proportion of unique genomes sequenced in the population. In this process, we used G matrix to calculate the genetic relationship between key individuals and the remaining population, details of the selection of key individuals in our study were described by Ye et al [11,12]. Finally, 21 males and 3 sires were selected as key individuals and re-sequenced with 150-bp paired-end reads on the Illumina HiSeq 3000 platform with the average sequence depth of 14.62. Sequencing reads were aligned to the *Gallus gallus* 4.0 genome using the Burrows-Wheeler Alignment tool [13]. Duplicated reads were removed using Picard release 1.119 (http://sourceforge.net/projects/picard/files/picard20tools/1.119/). GATK software [14] was used for SNP calling with the default parameters. Finally, 11,645,758 SNPs remained for further analysis and the concordance between chip data and sequence data was 98.0%. After that, the SNP chip was imputed to sequence data using Fimpute software [15] with key sequenced individuals as the reference. For more details about the genotype imputation, please refer to Ye et al [12]. Quality control both for the chip and imputed sequences genotypes were conducted use PLINK 1.90 software [16] with the same criteria: SNP call rates >0.97, minor allele frequencies >0.05, obeys Hardy-Weinberg equilibrium (p>0.00001), and individual call rate >0.95. After quality control, 447,766 SNPs from SNP chip and 8,626,020 SNPs from imputed sequences remained for subsequent analysis. These SNPs distributed on autosome with the average distance between adjacent SNPs ranged from 92.63 to 168.27 bp among different autosomes.

### Statistical analysis

Mixed linear model was used for the GWAS, and the analysis was performed with GEMMA software (Zhou and Stephens [17]). The statistical model is described as follows:

Y=Xb+Sa+Zu+e

where **Y** is the vector of phenotypic values for all individuals, **b** is a vector of fixed effects including the batch effect, which has three levels, **a** is the substitution effect of the SNP under consideration, **u** is the random additive genetic effect; X and Z are the design matrices for **b** and **u**, respectively; **S** is the design vector for a; **e** is the vector of random residuals. In this model, **u** and **e** were assumed to have the structure $u\sim N(0,G\sigma_{a}^{2})$ and $e\sim N(0,I\sigma_{e}^{2})$, where **G** is the additive genomic relationship matrix which was derived from the sequence data, $\text{G}=\frac{1}{p}\sum_{i=1}^{p}(X_{i}-I_{n}{\bar{X}}_{i}){\left(X_{i}-I_{n}{\bar{X}}_{i}\right)}^{T}$, **X** is the n×p matrix of genotypes of n individuals’ p markers, *X_i_* as its ith column representing genotypes of ith SNP *X̄_i_*, as the sample mean and *I_n_* as a n×1 vector of 1’s, $\sigma_{a}^{2}$ is the polygenic additive variance, I is an identity matrix and $\sigma_{e}^{2}$ is the residual variance. To compare the GWAS using the sequence data with chip data, the GWAS was performed using the chip data with the same model. DMU software [18] was used to estimate the heritability explained by the sequence data with the same model as the one described above.

To visualize the result from the GWAS, Manhattan plots and quantile-quantile plots were drawn by the qqman package [19] in R for each trait. In this study, the significant level was adjusted based on the effective number of independent tests, which was calculated by the simpleM software [20].

Candidate genes which were located within or nearby the significant SNPs (within 0.3 Mb) were identified by Ensembl (http://www.ensembl.org/index.html) and NCBI (https://www.ncbi.nlm.nih.gov/) annotation of the *Gallus gallus* 4.0 genome version.

### Comparing significant regions with reported quantitative trait loci

SNPs with p-value <1.0×10^−5^ were compared with all reported QTLs that associated with FCR. The QTLs were obtained from the Chicken QTLdb (https://www.animalgenome.org/cgi-bin/QTLdb/GG/index). Based on the physical location, QTLs close to the significant SNPs were extracted, with a maximum distance of 5 Mb between the QTL and the SNP to be compared.

## RESULTS

### Phenotype statistics and analysis of genetic parameter

Descriptive statistics for trait FCR are presented in Table 1. We found that the phenotypes of FCR were normally distributed (Shapiro-Wilk test, p>0.05), and its heritability was 0.33.

### Association results

The effective number of independent tests was 631,181 as calculated by the simple method. Hence, the threshold p-value was adjusted to 7.92×10^−8^ (0.05/631,181) for a genome-wide significance level, and to 1.58×10^−6^ (1.00/631,181) for a genome-wide suggestive significance level.

**Figure 1:**
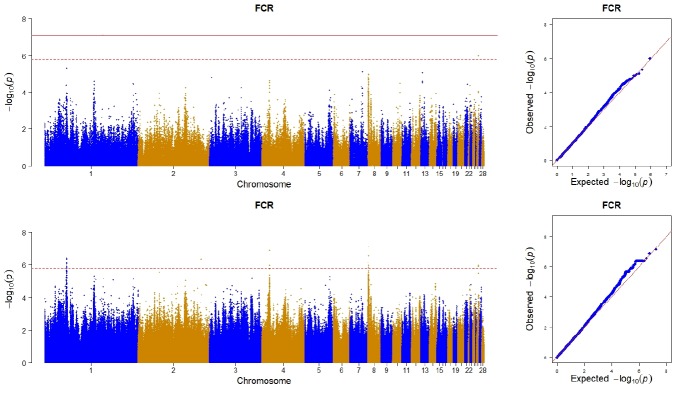
Manhattan plot (left) and quantile-quantile plot (right) of the observed p-values for feed conversion ratio (FCR) using chip data (top) and imputed whole sequence data (bottom). In the Manhattan plots, the position of each single nucleotide polymorphism on the chromosome was plotted against its −log_10_- transformed p-value. The dotted red line and solid red line in the Manhattan plots represent the significant threshold of 1.58×10^−6^ and 7.92×10^−8^ respectively. For quantile-quantile plot, the x-axis represents the expected −log10-transformed p-values, and the y-axis represents the observed −log10-transformed p-values.

In the case of using imputed whole sequence, one genome- wide significant SNP on chromosome 8 locating at 5th intron of zinc finger and BTB domain containing 41 (*ZBTB41*) was found to be associated with FCR, and 9 SNPs reached the suggestive significance level (Table 2). Among these 9 SNPs, 3 of them were located between 45.43 Mb and 45.61 Mb of chromosome 1 with the nearest genes being ubiquitin specific peptidase 44 (*USP44*), leukotriene A4 hydrolase (*LTA4H*), and ETS transcription factor (*ELK3*), respectively. One SNP was located on chromosome 2 and 60 Kb distance away from its nearest R-spondin 2 (*RSPO2*) gene. Two SNPs were located on a narrow region of chromosome 4 (from 15.59 Mb to 16.04 Mb), and the nearest genes were inhibitor of apoptosis protein 3 (*IAP3*) and sosondowah ankyrin repeat domain family member D (*SOWAHD*). Another two SNPs were located on the positions of 1.39 Mb and 2.71 Mb of chromosome 8 respectively, with the first SNP locating on the 14th intron of calmodulin regulated spectrin associated protein family member 2 (*CAMSAP2*) and the other locating on the 4th intron of potassium sodium-activated channel subfamily T member 2 (*KCNT2*). The remaining 1 SNP was located on the position of 3.20 Mb of chromosome 26 and member of RAS oncogene family (*RAP1A*) was its nearest gene. In the case of using chip data, only one SNP reach a suggestive significance level, and the SNP was also detected by using imputed whole sequence (Figure 1).

### Comparing significant regions with reported quantitative trait loci

**Figure 2:**
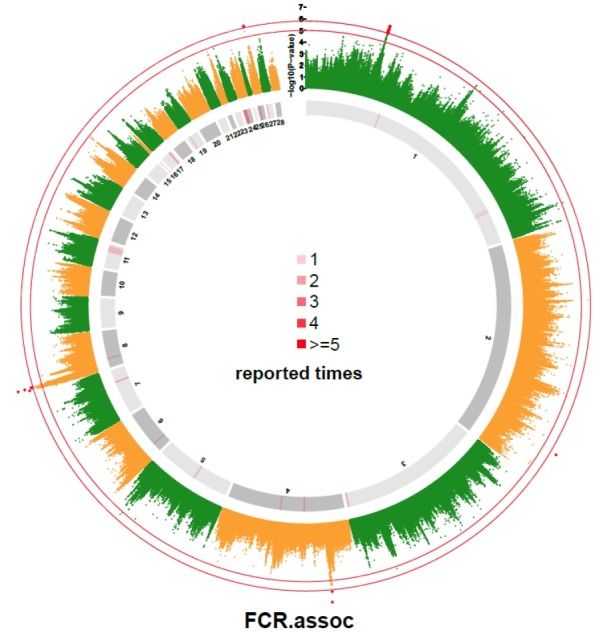
Genome-wide association study (GWAS) result of feed conversion ratio compared with reported quantitative trait loci (QTLs) associated with feed conversion ratio (FCR). The inner and outer circles were used to indicates the reported QTLs obtained from AnimalQTL database and from this study, respectively. The different shades of grey in the inner circle were used to distinguish the adjacent chromosomes. The red lines on the inner circle indicate the times of reported QTLs at the corresponding genome region. The outside circle is a Manhattan plot of the FCR with two thresholds at (1.58×10^−6^) and 5.0.

In our study, p-value of 31 SNPs was smaller than 1.0×10^−5^. They were compared with the reported QTLs collected from Chicken QTLdb. The results are shown in Supplementary Table S1. A total of 32 QTLs on autosomes were reported to be associated with FCR, and 8 of those QTLs were found near the 31 SNPs (Supplementary Table S1). Besides, seven SNPs located on chromosome 1 and 2 located far from reported QTL. Three out of the 7 SNPs located on a narrow region from 102.70 Mb to 106.46 Mb. The distribution of chicken FCR related QTLs as compared with SNPs with p-value lower than 1×10^−5^ are graphically displayed in Figure 2.

## DISCUSSION

Feeding traits are economically important in chicken industry, as they largely determine the edible percentage of the chicken and display moderate to high heritability. In this study, with the scientific feeding management and systematic record of phenotypes, a total of 435 birds’ average daily feed intake and average daily gain were used to calculate the FCR. Through the statistical test, the FCR presented the normal distribution (Shapiro-Wilk test, p>0.05). The hereditability estimates for FCR were higher than the estimate of Aggrey et al [21]. Based on the genetic parameter estimates, exploring the genetic mechanisms and identifying major genes would be useful for improving feed efficiency. In our study, we performed GWAS for FCR in a Chinese indigenous chicken population using both imputed whole sequence data and chip data.

To our expectation, the imputed sequence data including more SNPs could capture more genetic variation and detect more signals than a SNP array. With the same statistical model and significance level for both datasets, the SNPs detected from imputed whole sequence data were more than that from the chip data. On one hand, this demonstrated the power of imputed whole sequence data. On another hand, the stronger associations with imputed genotypes were partly due to some imputation artefacts driven by allele frequencies in the imputed loci.

In this study, the ten candidate genes detected for FCR are partly functionally related to feeding traits. Functional study showed that USP44 prevents the premature activation of the anaphase-promoting complex and regulating centrosome separation, positioning, and mitotic spindle geometry. LTA4H is an enzyme that would generate leukotriene B4 (LTB4) [22]. LTB4 is an extremely pro-inflammatory lipid mediator that can exert its activity by binding to receptors BLT1 or BLT2 [23]. ELK3 is a ternary complex factor and transcription repressor, which belongs to the ETS family involved in angiogenesis during embryonic development [24]. In addition, individuals lacking ELK3 protein had smaller tumors due to their inability to become vascularized and oxygenated. RSPO2 is a member of R-spondin family of proteins. These proteins are secreted ligands of leucine-rich repeat containing G protein-coupled receptors that enhance Wnt signaling through the inhibition of ubiquitin E3 ligases [25]. IAP3 encodes a protein that belongs to a family of apoptotic suppressor proteins. This protein functions through binding to tumor necrosis factor receptor-associated factors TRAF1 and TRAF2 and inhibits apoptosis induced by menadione, a potent inducer of free radicals, and interleukin 1-β converting enzyme [26]. *SOWAHD* is a protein coding gene linked to Iroquois genes, suggesting that regulatory constraints underlie the maintenance of the Iroquois-Sowah syntenic block [27]. CAMSAP2 specifically binds the minus-end of non-centrosomal microtubules, which can regulate the dynamics, organization, and polymerization of microtubules [28,29]. *ZBTB41* is a protein gene, which may be involved in transcriptional regulation [30]. *KCNT2* is a human gene that encodes the K_Na_ protein potassium channel activated by internal raises in sodium or chloride ions [31]. RAP1A encodes a member of the Ras family of small GTPases. The encoded protein undergoes a change in conformational state and activity, depending on whether it is bound to guanosine triphosphate (GTP) or guanosine diphosphate. This protein is activated by several types of guanine nucleotide exchange factors, and inactivated by two groups of GTPase-activating proteins [32].

QTLs that reported to be associated with chicken FCR are distributed across the whole genome. Part of them were reported several times in previous studies and overlapped with significant SNPs detected in this study, which suggested that the candidate regions detected in this study were reliable. Other SNPs that reached the suggestive significant levels were not located in QTL region may be the new candidate loci or statistically false positive signals.

The selection of significant level was seriously considered in this study. Though Bonferroni correction is widely used, it is rigorous in the genotype data with high linkage disequilibrium. Especially for such a high density imputed sequence data, the false negative results are not neglectable [33]. Hence, the number of effectively independent tests through principal component analysis [20] were used in this study.

## CONCLUSION

In this study, we conducted a genome wide association study for FCR using both imputed whole sequence data and SNP array in a Chinese indigenous chicken population. A list of significant SNPs and ten candidate genes were identified, and several of these regions were overlapped with QTLs reported in previous studies. Results from this study provided valuable prior information for chicken genomic breeding program and would potentially improve our understanding of the molecular mechanism for feeding traits.

## Supplementary Data



## Figures and Tables

**Table 1:** Descriptive statistics for phenotypes

Trait	N	Mean (SD)	Min	Max	CV (%)	h^2^(se)1)
FCR (%)	435	3.94±0.49	2.9	6.92	12.44	0.33 (0.11)

SD, standard deviation; CV, coefficient of variation; se, standard errors; FCR, feed conversion ratio; SNP, single nucleotide polymorphism.

1) Heritability and standard error estimated by DMU software package based on SNP genotypes.

**Table 2:** SNPs suggestive significantly associated with feed conversion ratio

Chr1)	Position	MAF	Beta2)	Se3)	−log10(P)	Near-gene	Distance (Kb)4)
1	45439962	0.053	0.36	0.07	5.92	*USP44*	U2.39
1	45582623	0.051	0.36	0.07	5.81	*LTA4H*	U1.72
1	45616618	0.053	0.38	0.07	6.40	*ELK3*	U5.16
2	131217033	0.051	0.36	0.07	6.34	*RSPO2*	U60.14
4	15596919	0.161	0.23	0.05	5.97	*IAP3*	U15.85
4	16040593	0.100	0.29	0.05	6.87	*SOWAHD*	U23.81
8	1395613	0.401	0.17	0.03	6.16	*CAMSAP2*	intro14
8	2583927	0.182	0.24	0.04	7.14	*ZBTB41*	intro5
8	2717880	0.181	0.22	0.04	5.97	*KCNT2*	intro4
26	3202060	0.374	0.18	0.04	5.98	*RAP1A*	U3.72

SNP, single nucleotide polymorphism; MAF, minor allele frequency; *USP44*, ubiquitin specific peptidase 44; *LTA4H*, leukotriene A4 hydrolase; *ELK3*, ETS transcription factor; *RSPO2*, R-spondin 2; *IAP3*, inhibitor of apoptosis protein 3; *SOWAHD*, sosondowah ankyrin repeat domain family member D; *CAMSAP2*, calmodulin regulated spectrin associated protein family member 2; *ZBTB41*, zinc finger and BTB domain containing 41; *KCNT2*, potassium sodium-activated channel subfamily T member 2; *RAP1A*, member of RAS oncogene family.

1) Chicken chromosome.

2) The effect size for marker.

3) The standard errors for effect.

4) D and U indicate the SNP is upstream and downstream of a gene, respectively.

## References

[1] Hume DA, Whitelaw CBA, Archibald AL (2011). The future of animal production: improving productivity and sustainability. J Agric Sci.

[2] Zhang W, Aggrey SE (2003). Genetic variation in feed utilization efficiency of meat-type chickens. World Poult Sci J.

[3] Havenstein GB, Ferket PR, Qureshi MA (2003). Growth, livability, and feed conversion of 1957 versus 2001 broilers when fed representative 1957 and 2001 broiler diets. Poult Sci.

[4] Rao Y, Shen X, Xia M (2007). SNP mapping of QTL affecting growth and fatness on chicken GGA1. Genet Sel Evol.

[5] van Kaam JB, Groenen MA, Bovenhuis H (1999). Whole genome scan in chickens for quantitative trait loci affecting growth and feed efficiency. Poult Sci.

[6] Abo-Ismail MK, Vander Voort G, Squires JJ (2014). Single nucleotide polymorphisms for feed efficiency and performance in crossbred beef cattle. BMC Genet.

[7] Onteru SK, Gorbach DM, Young JM (2013). Whole genome association studies of residual feed intake and related traits in the pig. PLoS One.

[8] Xu Z, Ji C, Zhang Y (2016). Combination analysis of genome-wide association and transcriptome sequencing of residual feed intake in quality chickens. BMC Genomics.

[9] Isotouru T, Sahana G, Guldbrandtsen B, Lund MS, Vilkki J (2016). Genome-wide association analysis of milk yield traits in Nordic Red Cattle using imputed whole genome sequence variants. BMC Genet.

[10] Zhang Z, Xu ZQ, Luo YY (2017). Whole genomic prediction of growth and carcass traits in a Chinese quality chicken population. J Anim Sci.

[11] Druet T, Macleod IM, Hayes BJ (2014). Toward genomic prediction from whole-genome sequence data: impact of sequencing design on genotype imputation and accuracy of predictions. Heredity (Edinb).

[12] Ye S, Yuan X, Lin X (2018). Imputation from SNP chip to sequence: a case study in a Chinese indigenous chicken population. J Anim Sci Biotechnol.

[13] Li H, Durbin R (2009). Fast and accurate short read alignment with Burrows-Wheeler transform. Bioinformatics.

[14] Mckenna A, Hanna M, Banks E (2010). The Genome Analysis Toolkit: a MapReduce framework for analyzing next-generation DNA sequencing data. Genome Res.

[15] Sargolzaei M, Chesnais JP, Schenkel FS (2014). A new approach for efficient genotype imputation using information from relatives. BMC Genomics.

[16] Purcell S, Neale B, Toddbrown K (2007). PLINK: a tool set for whole-genome association and population-based linkage analyses. Am J Hum Genet.

[17] Zhou X, Stephens M (2012). Genome-wide efficient mixed-model analysis for association studies. Nat Genet.

[18] Madsen P, Sørensen P, Su G (2014). DMU - a package for analyzing multivariate mixed models.

[19] Turner SD (2014). qqman: an R package for visualizing GWAS results using Q-Q and manhattan plots. Biorxiv.

[20] Gao X, Starmer J, Martin ER (2008). A multiple testing correction method for genetic association studies using correlated single nucleotide polymorphisms. Genet Epidemiol.

[21] Aggrey SE, Karnuah AB, Sebastian B, Anthony NB (2010). Genetic properties of feed efficiency parameters in meat-type chickens. Genet Sel Evol.

[22] Fuchs G, Shema E, Vesterman R (2012). RNF20 and USP44 regulate stem cell differentiation by modulating H2B monoubiquitylation. Mol Cell.

[23] Low CM, Akthar S, Patel DF (2017). The development of novel LTA4H modulators to selectively target LTB4 generation. Sci Rep.

[24] Rogers CD, Phillips JL, Bronner ME (2013). Elk3 is essential for the progression from progenitor to definitive neural crest cell. Dev Biol.

[25] Ilmer M, Recio BA, Regel I (2015). RSPO2 enhances canonical Wnt signaling to confer stemness-associated traits to susceptible pancreatic cancer cells. Pancreatology.

[26] Pachlopnik SJ, Canioni D, Moshous D (2011). Clinical similarities and differences of patients with X-linked lymphoproliferative syndrome type 1 (XLP-1/SAP deficiency) versus type 2 (XLP-2/XIAP deficiency). Blood.

[27] Maeso I, Irimia M, Tena JJ (2012). An ancient genomic regulatory block conserved across bilaterians and its dismantling in tetrapods by retrogene replacement. Genome Res.

[28] Jiang K, Hua S, Mohan R (2014). Microtubule minus-end stabilization by polymerization-driven CAMSAP deposition. Dev Cell.

[29] Hendershott MC, Vale RD (2014). Regulation of microtubule minus-end dynamics by CAMSAPs and Patronin. Proc Natl Acad Sci USA.

[30] Yokoyama S, Ito Y, Ueno-Kudoh H (2009). A systems approach reveals that the myogenesis genome network is regulated by the transcriptional repressor RP58. Dev Cell.

[31] Santi CM, Ferreira G, Yang B (2006). Opposite regulation of Slick and Slack K+ channels by neuromodulators. J Neurosci.

[32] Sayyah J, Bartakova A, Nogal N (2014). The Ras-related Protein, Rap1A, mediates thrombin-stimulated, integrin-dependent glioblastoma cell proliferation and tumor growth. J Biol Chem.

[33] Lautenberger JA, Troyer JL, Nelson GW (2010). Accounting for multiple comparisons in a genome-wide association study (GWAS). BMC Genomics.

