# Supplementary material for: Genome wide association study on feed conversion ratio using imputed sequence data in chickens
Source: Asian-Australas J Anim Sci. 2018 Oct 26;32(4):494–500. doi: 10.5713/ajas.18.0319 (PMC6409457; doi:10.5713/ajas.18.0319)
Supplement: Supplementary file 1 [file ajas-18-0319-supplementary.pdf]

Additional file

Table S1. SNPs distribution in QTL associated with FCR

Chr[a]	Position	-log10(P)	Candidate	Dis(Kb)[b]	QTL	Dis(Mb)[c]
1	45294365	5.23	FGD6	intro19	33.28- 47.37	within
1	45352736	5.66	VEZT	intro11	33.28- 47.37	within
1	45439962	5.92	USP44	U2.389	33.28- 47.37	within
1	45471662	5.68	NTN4	intro1	33.28- 47.37	within
1	45582623	5.81	LTA4H	U1.72	33.28- 47.37	within
1	45616618	6.40	ELK3	U5.164	33.28- 47.37	within
1	102706431	5.18	ADAMTS5	U263.685	NA	NA
1	103029989	5.32	uc_338	U197.969	NA	NA
1	106462834	5.10	DSCR3	U5.608	NA	NA
1	123749382	5.19	ARHGAP6	intro9	NA	NA
1	142580034	5.08	FGF14	intro3	NA	NA
1	185326516	5.14	SLC36A4	intro8	NA	NA
2	44336526	5.54	TRAK1	intro2	NA	NA
2	99687785	5.05	ARHGAP28	U110.023	NA	NA
2	131217033	6.34	RSPO2	U60.141	NA	NA
3	89741021	5.20	CSMD1	intro58	NA	NA
4	15549128	5.38	STAG2	intro13	18.24- 31.13	D2.7
4	15596919	5.97	IAP3	U15.85	18.24- 31.13	D2.65
4	16040593	6.87	SOWAHD	U23.813	18.24- 31.13	D2.2
5	51216482	5.07	TMEM179	U91.307	NA	NA
5	51600899	5.30	ZBTB42	U1.25	NA	NA
8	1395613	6.16	CAMSAP2	intro14	6.97- 7.84	D5.57
8	1648014	5.45	NR5A2	intro3	6.97- 7.84	D5.32
8	1839885	5.28	gga-mir-181a-1	U146.801	6.97- 7.84	D5.13
8	2123107	5.14	ATP6V1G3	U12.048	6.97- 7.84	D4.84
8	2273294	5.37	LHX9	intro2	6.97- 7.84	D4.69
8	2583927	7.14	ZBTB41	intro5	6.97- 7.84	D4.38
8	2717880	5.97	KCNT2	intro4	6.97- 7.84	D4.25
8	3155433	5.10	CDC73	U229.314	6.97- 7.84	D3.81
26	3202060	5.98	RAP1A	U3.716	0.06- 2.54	D0.66
26	3204278	5.49	FAM212B	U5.055	0.06- 2.54	D0.67

[a]chicken chromosome.

[b] D and U indicate the SNP is upstream and downstream of a gene, respectively.

[c] D and U indicate the SNP is upstream and downstream of a QTL, respectively.
